# Imagining and Preventing the Future Existence of Bodies with Variations in Sex Characteristics

**DOI:** 10.1007/s10508-025-03261-9

**Published:** 2025-11-18

**Authors:** Limor Meoded Danon, Tamar Paperna, Hagit Daum, Shachar Zuckerman

**Affiliations:** 1 The Azrieli Faculty of Medicine, Bar-Ilan University, Henrietta Szold St 8, 1311502 Safed, Israel; 2 Rambam Health Care Campus, Haifa, Israel; 3 Center for Clinical Genetics Hadassah, Jerusalem, Israel; 4 Genetic Unit, Shaare Zedek Medical Center, Jerusalem, Israel

**Keywords:** Variations in sex characteristics, Disorders of sex development, Preimplantation genetic testing, Genetic counseling, Fertility

## Abstract

The diagnosis of bodies with variations in sex characteristics (VSC) is a powerful social act that affects the social realities of patients, doctors, and families. This process includes an imagined connection between various biological characteristics and sociocultural norms of gender and fertility. We studied how Israeli medical professionals approach VSC in fertility and prenatal settings. Our research considered two contrasting trends: the growing recognition of VSC individuals’ rights to make decisions about their bodies and the increasing use of genetic testing to prevent VSC conditions. We conducted in-depth interviews with 27 biomedical professionals from different hospitals in Israel and collected data on preimplantation genetic testing cycles at three central hospitals in the country to identify VSC genetic conditions. The findings show how tensions and gaps regarding perceptions of fertility and bodies with diverse sex development exist among various specialists and their patients across different clinical interactions. We describe these gaps and their physical and social outcomes through three themes: “controlling the diagnostic emergency of VSC,” “secrets and concealment in the genetic diagnostic process,” and “imagining and preventing the existence of fertile bodies.”

## Introduction

Diagnosis is a powerful social practice that authorizes biomedical professionals’ classification and categorization of symptoms and social behaviors that are considered deviant (Brown, 1990, 1995; Conrad & Schneider, 1992; Jutel, 2009). Sociologists examine how diagnosis shapes experiences of illness and determines which voices are privileged or silenced (Jutel, 2015, p. 845). The term “diagnostic imaginary” describes “a way of thinking which conceals the presence of the social in diagnoses, closing off critical analysis of the complex ways they are structured by history, culture, politics, and value judgments” (McGann, 2011, p. 337). The diagnostic process is a locus of sociopolitical interests where various stakeholders, including lay people, social groups, pharmaceutical corporations, patient groups, and biomedical professionals—collaborate and clash (Conrad, 2005; Jutel, 2014; Payer, 1992).

Studies have shown how the diagnostic process gives biomedical professionals control over people’s bodies and experiences (Jutel & Buetow, 2007), increases the number of pharmaceutical customers (Conrad & Leiter, 2008; Ebeling, 2011), helps patients gain social-familial-occupational recognition and legitimation (Charmaz, 1991; Zavestoski et al., 2004), changes medical policies to improve treatment (Epstein, 1996; Rosenberg, 2006), and, especially relevant for LGBTQI groups, changes in diagnostic criteria (e.g., removing homosexuality from the DSM in 1973; the term “gender dysphoria” replaced the term “gender identity disorder” in the DSM-5) and medical practices (Davis, 2011; Eckhert, 2016). The sociology of diagnosis rests on the assumption that what medical practitioners seek to diagnose, or what is perceived as a “disorder,” “abnormal,” or “atypical,” is socially constructed. As Conrad and Barker (2010) explain:

First, some illnesses are particularly embedded with cultural meaning, which is not directly derived from the nature of the condition—which shapes how society responds to those afflicted and influences the experience of that illness. Second, all illnesses are socially constructed at the experiential level, based on how individuals come to understand and live with their illness. Third, medical knowledge about illness and disease is not necessarily given by nature but is constructed and developed by claims-makers and interested parties. (p. 67)

The term “diagnostic imaginary” (McGann, 2011, p. 337) invites us to consider the diagnostic process a liminal space where unique moments and interactions exist and the lines between the imagined and the real, the actual and the abstract, and the certain and uncertain intersect. Uncertainty and liminal spaces are integral aspects of the diagnostic process of variations in sex characteristics (VSC). We use both terms—intersex and VSC—to describe bodies that differ genetically, in terms of their gonads or genitals, and in other ways—from typical, normative male or female bodies. Intersex and VSC represent bodies in a liminal space regarding knowledge, perceptions, and actions and between what is known and unknown about sex development and gender assignment (Fausto-Sterling, 2012; Richardson, 2013).

Intersex and VSC bodies are caught between binary thinking and cultural expectations about human bodies and the practical outcomes of the diagnosis and treatment of female and male bodies (Carpenter, 2018; Domurat-Dreger, 1998; Friedman, 2013; Kessler, 1998), between definitions and categorizations continuously changed by activists, medical professionals, and scholars, between pathological and sociocultural norms, and between linguistic terms such as hermaphrodite, intersex, disorders or differences of sex development (DSD), and VSC. The current medical classification includes three subcategories under DSD (46XX DSD, 46XY DSD, and sex chromosome DSD). These subcategories highlight the dominance of sex karyotype and genetics in the diagnosis process (Conway, 2022; Röhle et al., 2017). Nevertheless, many conditions remain unclassified (Délot & Vilain, 2021; Granada & Audi, 2021). Naming and classifying VSC leads to sex/gender assignments, especially in cases of atypical genitalia (Byers et al., 2020), as well as surgical genital interventions that help parents and future patients adjust to (binary) sociocultural norms (Creighton et al., 2014; Sanders et al., 2012).

The diagnostic process leads to a chain of actions and decisions to preserve the physical-gender norm and also creates pathological living experiences, such as living with a toxic secret that creates alienation, loneliness, and depression (Davis, 2015; Frank, 2018; Jones et al., 2016; Meoded Danon, 2015, 2022; Preves, 2003; Roen & Lundberg, 2020); living with trauma, shame, and rage (Hart & Shakespeare-Finch, 2022); and a long-term search for home and stability (Zieselman, 2020).

Fertility and the meaning of parenthood constitute significant issues in the chain of implications stemming from the diagnostic process of VSC. Biological parenthood is a priority and concern for parents of children/adults with VSC and individuals born with VSC (Johnson et al., 2017; Słowikowska-Hilczer et al., & dsd-LIFE Group, 2017). A recent study found that “while discussing fertility may be difficult initially, it is better done sooner rather than later as adjustment and coping improve with time” (Papadakis et al., 2021). Other studies highlight fertility and ethical issues relevant to young adults with VSC, such as unknown medical/biological factors and their potential impact on fertility, the timing of treatments and surgeries, experimental procedures, and the transmission of the medical condition (Campo-Engelstein et al., 2017; Słowikowska-Hilczer et al., & dsd-LIFE Group, 2017). Jones describes her participants’ coping with infertility as encompassing “a sense of loss,” the need to find ways to adjust to a new path in their lives, bearing the burden of early diagnosis, and embracing the positive aspects of being child-free (Jones, 2020).

In examining the practices and meanings of the VSC diagnostic process, it is important to consider two concurrent processes occurring globally and locally. The first is the international intersex movement’s promotion of legislation to protect bodily integrity and human rights (Bauer et al., 2020; Carpenter, 2022). Notably, especially today, when democracy and human rights are fragile, intersex human rights can be achieved only in strongly democratic countries where human rights laws are valued and protected. The second process is the geneticization of sex development and VSC, which includes attempts to reveal the genetic etiology of VSC conditions (Ahmed et al., 2022; Délot & Vilain, 2021; Eozenou et al., 2020; Gonen & Lovell-Badge, 2019). On the one hand, this process can further patients’ and medical professionals’ understanding of their physical development and health issues and assist them in making decisions that affect their and their children’s futures (Meoded Danon, 2021).

On the other hand, however, discoveries of genetic etiologies may threaten the existence of fetuses diagnosed early with VSC. Studies point to a high frequency of termination of pregnancy (TOP) following the diagnosis of sex chromosome aneuploidy (Bevilacqua et al., 2018; Jeon et al., 2012; Sagi et al., 2001; Shaffer et al., 2006). The main reasons for termination are reported to be related to parental fear and anxiety, infertility issues, and directive counseling (Jeon et al., 2012). Moreover, the geneticization of VSC also leads to the use of preimplantation genetic testing (PGT) and embryo selection. We elaborate on this subject in the next section.

How do geneticists, genetic counselors, and fertility experts in different Israeli hospitals perceive and implement practices aimed at preventing VSCs? In this article, we explore how the concept of “the diagnostic imaginary” is manifested in the context of genetic counseling for VSC fertility issues and during various clinical encounters and interactions with people with VSC. We begin by briefly describing the dynamic relationship between reprogenetic technologies and VSC.

### Between Reprogenetic Technologies and Bodies with Variations in Sex Characteristics

How people think about reproduction comprises an integral part of how they do it: human reproduction does not simply happen by itself, just as there is no “automatic” biological mechanism that produces new human beings (Franklin, 2013, p. 748).

The medical consensus regarding the importance of early diagnosis of intersex/VSC and the development of reprogenetic technologies have increased the prenatal diagnosis of infants and embryos with these conditions. The most widely used genetic technology is noninvasive prenatal testing (NIPT), which involves the analysis of cell-free fetal DNA in maternal blood from as early as seven to ten weeks of pregnancy (Pinti et al., 2022). The time of diagnosis is critical for several reasons. First, early diagnosis prepares medical professionals and parents for the future child’s needs and treatment possibilities. Early diagnoses can save the lives of babies born with classic congenital adrenal hyperplasia (CAH) with hormonal and salt imbalances (Simpson & Rechitsky, 2019). Since 2008, neonatal screening for the diagnosis of CAH has taken place in Israel (Raz et al., 2019), and according to the Ministry of Health, approximately eight to nine children with CAH are born per year (Ministry of Health, personal communication, October 22, 2023). Second, prenatal diagnosis enables people to exercise their reproductive rights and autonomously decide whether, when, and under what circumstances to become parents (Vainik, 2008). Third, early prenatal diagnoses may lead to negative consequences, including pressure on parents, especially mothers, to terminate pregnancies or undergo PGT to detect and select embryos (Holmes, 2008).

PGT is conducted following IVF when a blastomere (single cell) is removed from an eight-cell embryo on the third day of fertilization. Alternatively, several cells of a five-day embryo can be biopsied and tested for specific variants related to a familial disease. As part of Israel’s pronatalist policy, the state subsidizes IVF cycles with PGT up to the birth of two babies. Scholars have addressed ethical and biopolitical issues related to PGT. These include human eugenics (Camporesi & Cavaliere, 2018; Efron & Lifshitz-Aviram, 2020; Raz et al., 2022), women’s reproductive choices (Murphy, 2012), and medical professionals’ and users’ assumptions that PGT will prevent future suffering (Zuckerman et al., 2017, 2020).

With the increasing global use of NIPT technology in the prenatal diagnosis of aneuploidies, identifying sex chromosome aneuploidies (SCAs) has become one of the reasons for prenatal testing and TOP (Johnston et al., 2023; Stevens et al., 2023). The main reasons for termination are lack of knowledge and support and anxiety regarding the offspring’s infertility (Jeon et al., 2012; Meoded Danon, 2018, 2022). These findings are limited because they refer mainly to secular parents in countries with less strict regulation of TOP and broader access to reprogenetic technologies than Israel. Moreover, SCAs comprise only a small portion of VSC conditions. Many other conditions related to gonadal development and genital formation are detected prenatally. Nevertheless, few studies have examined pregnancy terminations due to other VSC conditions, such as classic/nonclassic congenital adrenal hyperplasia, partial/complete androgen insensitivity syndrome, Swyer syndrome, 5-alpha-reductase deficiency, and 17-beta-hydroxysteroid dehydrogenase deficiency.

In the geneticization process of VSC, next-generation sequencing has become the dominant tool for genetic diagnosis and the expansion of the gene pool associated with VSC conditions (Aekka et al., 2024; Délot & Vilain, 2021; Ea et al., 2021). These practices established the foundation for the use of PGT to detect and prevent VSC genetic conditions following IVF and before pre-embryo implantation. Studies show that physicians, geneticists, and parents perceive PGT positively and prefer this procedure over TOP (Meoded Danon, 2022; Zuckerman et al., 2020). Nevertheless, no studies have explored the use of PGT to prevent VSC conditions. In Jones and colleagues’ online survey of 170 intersex people from Australia, 81% (137/170) of participants disagreed (or strongly disagreed) with the suggestion that “people should select against having intersex offspring (e.g., by using IVF selection techniques),” 4% (7/170) agreed (or strongly agreed), and 8% (14/170) were neutral or unsure (Jones et al., 2016, p. 195). However, this survey is not sufficiently comprehensive to reflect the diversity of perspectives on preventive practices. It would be interesting to examine the differences among individuals with various conditions and explore how cultural and familial factors influence their decision making.

In the last decade, new reproduction practices and techniques that include fertility preservation, egg freezing, uterus transplantation, and testicular sperm retrieval (TESE/micro TESE) have significantly influenced fertility among people with VSC conditions. PGT may be a significant part of the fertility regulation process, especially in countries with no regulations or soft regulations regarding the conditions under which it should be used, such as Israel, the USA, Australia, Mexico, China, Japan, and others (Ginoza & Isasi, 2020). This study’s participants encounter couples and families of children and adults with intersex/VSC and people with intersex/VSC who are undergoing various fertility processes, including genetic counseling for PGT. We describe and explore their perspectives and diagnostic practices.

## Method

This article is part of a broader, ongoing qualitative and quantitative international study examining the dynamics between prevention and inclusion practices aimed at intersex/VSC bodies in various fertility processes from different perspectives. The research explores the use and outcomes of reproduction technologies, examining the experiences of the actors in this field and the dynamics and relationships between them.

### Participants

To learn about biomedical professionals’ perspectives and experiences with VSC and PGT, we conducted 27 semi-structured interviews, in different time frames, with geneticists, gynecologists, genetic counselors, endocrinologists, and a social worker at several fertility and genetics units in Israel. (Participant details appear in Tables 1 and 2) For the first round of interviews, the participants were recruited by direct emails (sent to relevant experts) that presented the research subject and questions and included the consent form. Of the 14 physicians approached, four did not respond. The co-authors recruited participants for the second round of interviews after receiving ethical approval from their hospitals.

**Table 1:** First round of interviews focusing on the use of preimplantation genetic testing to prevent variations in sex characteristics

No	Pseudonym	Profession	Date	Method, Location
1	Prof. Vered	Pediatric geneticist	22.2.18	Face to face (FTF), Office
2	Prof. Ada	Pediatric geneticist	8.3.18	FTF, Office
3	Prof. Bery	Pediatric geneticist	25.3.18	FTF, Office
4	Dr. Kiperman	Gynecologist/ Geneticist	15.4.18	FTF, Office
5	Dr. Morag	Gynecologist/ Fertility specialist	17.11.19	FTF, Office
6	Prof. Dagan	Pediatric/ Endocrinologist	24.11.19	FTF, Office
7	Prof. Lorry	Gynecologist/ Fertility specialist	2.12.19	FTF, Office
8	Prof. Bracha	Gynecologist/ Fertility specialist	8.12.19	FTF, Office
9	Prof. Lerner	Gynecologist/ Fertility specialist	11.12.19	FTF, Office
10	Dr. Josef	Gynecologist/ Geneticist	17.2.20	FTF, Office

**Table 2:** Second round of interviews on the use of preimplantation genetic testing to prevent variations in sex characteristics

No	Pseudonym	Profession	Date	Method, Location
11	Galit	Genetic counsellor	11.7.21	FTF, Office
12	Dr. Yuval	Pediatric geneticist	11.7.21	FTF, Office
13	Maayan	Genetic counsellor	11.7.21	FTF, Office
14	Prof. Nava	Gynecologist/ Geneticist	18.7.21	FTF, Office
15	Dr. Roni	Gynecologist/ Fertility specialist	9.8.21	Zoom
16	Dr. Aki	Gynecologist/	19.1.22	Zoom
		Obstetrician		
17	Prof. Hanan	Gynecologist/	10.3.22	FTF, Office
		Obstetrician		
18	Dr. Shay	Genetic counsellor	31.5.22	FTF, Office
19	Dr. Tal	Genetic counsellor	31.5.22	FTF, Office
20	Prof. Gil	Pediatric endocrinologist	15.6.22	Zoom
21	Dr. Felix	Pediatric geneticist	19.6.22	Zoom
22	Dr. Shery	Gynecologist/Geneticist	20.6.22	FTF, Office
23	Amit	Genetic counsellor	20.6.22	FTF, Office
24	Dr. Rim	Gynecologist/Geneticist	26.6.22	Zoom
25	Dr. Danilovich	Pediatric Geneticist	4.8.22	Zoom
26	Rivi	Social worker	1.9.22	Zoom
27	Dr. Dalit	Gynecologist/Geneticist	8.9.22	Zoom

### Procedure and Measures

The interviews were conducted at different times due to transitions between academic institutions. In the first phase, the first author was completing her post-doctoral research at another university, and this transition necessitated a pause in the research, alongside attempts to obtain data from the Ministry of Health on the prevalence of babies with VSC conditions, TOP, and the use of PGT for congenital adrenal hyperplasia and androgen insensitivity syndrome, for example. The Israeli Ministry of Health has no registry of VSC diagnoses. One reason for this lack of data is that doctors are not obligated to report on this diagnosis or TOPs (before the twenty-third week of gestation) following a prenatal diagnosis of VSC. There is currently no national registry of babies born with VSC conditions. Hence, Meoded Danon sought to collaborate with geneticists from central hospitals in Israel that commonly practice PGT. We applied for institutional review board approval at each hospital. This process took approximately two years. This approval enabled us to access relevant data files through the hospitals’ databases and contact other relevant medical professionals at each hospital. Obtaining data on VSC from different units (i.e., gynecology, genetics, endocrinology) required considerable coordination and cooperation. In many instances, the units do not systematically record VSC cases. They use different computer programs, and cases have been documented only for the years since the programs began to be used. Moreover, doctors may use different terms and codes (ICD9, ICD10, and others) to register patients with VSC. We managed to obtain some of these data related to prenatal diagnosis and diagnosis at birth and later in life from only one of three hospitals. Nevertheless, surprisingly, as mentioned in many interviews, after prenatal diagnosis, which often occurs in this public hospital, the unofficial policy is not to perform pregnancy terminations, which are legal in Israel (after approval by a dedicated committee until the 24th week, and with more severe restrictions beyond that point). Parents who want a selective abortion are referred to another hospital in the same area. This sensitive information makes data collection and cross-referencing from different units and hospitals more difficult. Therefore, throughout the lengthy data collection process, we were aware that we could not obtain a comprehensive picture of VSC cases from these hospitals, especially at different stages of the diagnostic process.

The interviews were conducted by Dr. Meoded Danon and held face to face or via Zoom (see Tables 1 and 2). The participants were asked general questions about their professional roles and their understanding of biological sex and its determination. More specifically, they were asked about their perspectives on VSC, prenatal diagnostic processes, and the use of PGT. The interview explored their significant interactions with VSC patients and the conflicts and challenges they encountered in their practices, especially regarding genetic diagnostic technologies used during and before pregnancy. The interview further addressed their views on the intersex rights movement, particularly regarding bodily autonomy and how it relates to the use of PGT.

### Data Analysis

We collected and analyzed the data based on six stages of thematic analysis (Clarke et al., 2015) and the grounded theory approach (Charmaz & Thornberg, 2021). Shortly after the completion of the first-round interviews, Meoded Danon coded and categorized the interviews’ topics and examined issues that arose with her co-authors. The next round of interviews examined and analyzed issues such as secrecy in genetic settings, dexamethasone use, and views on TOP.

From the available data, we sought to determine how many PGT consultations regarding VSC were conducted during the last decade, how many preimplantation genetic tests were carried out in the context of VSC, why, and what the consequences were, i.e., how many pregnancies resulted and how many babies were born. We obtained data from two genetic units. The third genetic unit could provide no information about PGT being performed to identify VSC. The data that were available and documented in the genetics units of the two hospitals did not include all the information we requested, and the two hospitals documented the data differently. For example, there was a lack of data on when the PGT was performed, how many embryos were diagnosed, and how many were implanted. Data on the continuation of the fertility treatments and reasons for not continuing them were unavailable. Moreover, due to the sensitivity and confidentiality of these data and the paucity of documented cases, we could not include even the general demographic characteristics (religion, ethnic background, area of residence) of each case.

However, we can state from the personal experience of the co-authors involved in the consulting process that in most cases of PGT use, the parents were ultra-Orthodox Jews. This finding corresponds with those of other studies. For example, Zuckerman et al. (2020) explain how “religion played a decisive role in accepting PGT as an antenatal option” in their study. Zalcberg-Block and Zalcberg (2024) describe the central role of Ashkenazi rabbis in promoting the routinization of premarital genetic testing in their communities. In the following section, we present the themes “controlling the diagnostic emergency,” “secrets and concealment in the genetic diagnostic process,” and “imagining and preventing the existence of fertile bodies” to explore how VSC conditions are diagnosed, perceived, and targeted by preventive practices.

## Results

### Controlling the Diagnostic Emergency of Variations in Sex Characteristics

This theme illustrates how genetic diagnostics are expected to control the social, cultural, and familial emergency surrounding babies’ atypical genitalia and the gap between this expectation and reality in different clinical interactions and settings in Israel.

There’s [general] agreement that [atypical genitalia] is urgent. It’s really urgent, less than 24 hours [after birth] … ICU-level urgency, just to tell parents something. It’s a boy. It’s a girl. What do we see? And all the pre-pregnancy screening of the couples, every moment a new test … It’s a completely different mindset ... It’s a race toward the truly perfect child, and … parents are not willing to accept anything else. But … of course, it happens [that a child is born with a physical difference]. Quite a few couples say, “It’s a blessing, and wow, how good it is that we have him, and what would our lives be like without him? But, come on, let’s say that until that moment, they do everything they can to prevent this [from happening].” (Prof. Nava)

Genetic testing has become a widespread and routine diagnostic practice due to the intensification and increasing dominance of the genetic discourse in medicine and the ongoing refinement of diagnostic technologies and methods. Prof. Nava, director of the genetics unit at a central hospital in Israel, highlighted various issues and contradictions inherent in the prenatal diagnostic process in the country. First, the stressful atmosphere of fertility-, pregnancy-, and birth-related clinical settings affects how medical professionals, patients, and parents perceive fetuses or embryos throughout the diagnostic process. The main reasons for this atmosphere are related to the attempt to diagnose genetic diseases as early as possible to prevent the occurrence or recurrence of genetic diseases. Second, the mutual influence of genetic technological tools, on the one hand, and the discovery of the genetic etiology of various diseases, on the other, are embodied in the multitude of diagnostic tests offered before, during, and after pregnancy. Third, the sense of taking part in a race “toward the truly perfect child” that Prof. Nava mentioned is part of a narrow mindset that limits our view of children’s bodies to genetic coding, gives excessive weight to genetic technologies as a source of physical truth, and delays carrying and interacting with new babies (which can be “a blessing,” as Prof. Nava explained).

The line between medical and social emergencies is blurry in the case of VSC. Atypical genitalia are significant in the context of the diagnosis of 46XX classic congenital adrenal hyperplasia, or 21-hydroxylase deficiency (21-OHD). Twenty-one-hydroxylase is an enzyme responsible for producing cortisol. Its absence or a genetic variant that affects its expression may create a chain reaction in hormonal balance that can involve low levels of cortisol, needed to deal with physical and emotional stress and maintain adequate energy supply and blood sugar levels; high levels of androgens that affect genital development and reproductive organs; and, in some cases, low sodium and potassium, which affect heart function (Tamhane et al., 2018). Dr. Dalit spoke of this blurry line in such cases:

I was called to see a baby born with a genital organ [but] without a scrotum, and the doctors couldn’t tell whether it was male or female. It wasn’t that the testicles were intact and the scrotum was empty; there was no scrotum (the ultra-Orthodox Jewish parents did not do testing during the pregnancy) … [It] seems very trivial [to] call your family, your parents, your siblings, and say, “Congratulations, we have a son.” They couldn’t do this because they weren’t sure what it was. This is a terrible predicament. ... I was asked to do [the consultation] urgently to understand whether there was an SRY [gene] and what it was.^1^ Was it an enlarged clitoris? … When you look at the genitalia and don’t know whether [the baby] is male or female, it’s considered an emergency because if it’s CAH, this baby will collapse shortly because of … salt wasting. So, okay, that puts aside for a moment the feelings of the parents who don’t understand what the baby was born with, which means that it needs to be treated urgently. Nevertheless, I don’t know what’s more urgent— the collapse of the child’s systems or the parents’ distress. (Dr. Dalit, gynecologist and geneticist)

Dr. Aki also described his difficulty with diagnosing atypical genitalia, especially immediately after birth, and explaining it to parents:

You know that sometimes they come out, and we don’t see. The baby is [born and]… I look at its genitalia and don’t know what to say … Sometimes a heart defect (I’m not joking) or a cleft lip is better because it’s a clear thing and people relate to it. Telling a woman I don’t know if it’s a boy or a girl breaks them … [because] they don’t know whether there will be a circumcision or not, or what to say. (Dr. Aki, OB-GYN)

The blurry line between medical and social emergencies that these quotes describe emphasizes the powerful meaning of genital appearance and its social function. Unlike parental fear of social stigma associated with deafness, albinism, or genetic diseases that put children’s lives at risk, the fear of atypical genitalia is deeply rooted in anxieties about sex-gender variance, social stigmatization of queer identities, and deviation from heteronormative life. In the Jewish-Israeli context, the way male genitalia is viewed cannot be separated from the sociocultural norm of foreskin removal in the religious circumcision ceremony (Meoded Danon, 2021). Thus, this gaze is culturally embedded in and affiliated with religion and community (Earp et al., 2023; Reis, 2013).

In addition, there is a space of interpretation in every interaction with bodies with VSC that moves and changes according to the timing of the diagnosis, parents’ experiences, specialists’ interpretations of the relationships between body, gender, and sexuality, the cultural-religious context, and other factors. Prof. Gil, one of the most experienced pediatric endocrinologists in Israel, emphasized how the right to bodily autonomy for VSC does not conform to Israeli religious norms:

The claim is always that maybe one day I’ll be late coming home, the neighbor will be watching the children, she’ll have to diaper her, and she’ll see what she has there and be frightened. This is a claim that probably wouldn’t be made in the United States because there’s simply no such thing as involving the neighbor in diapering the children ... Yesterday, I worked at a clinic in the ultra-Orthodox sector. I noticed that three families of the seven children I saw had 14 children. In such families, there is no time for all the philosophical discussions. You need a girl or a boy, and we are done [laughs], and [this attitude] also has advantages. You don’t drive the child crazy. You don’t disturb him mentally. He knows he’s a boy. She knows she’s a girl. At some point, they’ll know if there is a fertility problem, and we’re done. Like a hundred years ago. It was simpler. (Prof. Gil, pediatric endocrinologist)

The alleged simplicity, as it exists in Prof. Gil’s view of ultra-Orthodox families regarding body-gender norms, is perceived as the opposite of the complex, nonbinary sex/gender view of intersex activists. Prof. Gil emphasizes how the binary view makes the treatment process easier for everyone, especially parents and doctors. However, he ignores the patient’s view and experience of binarism, especially within ultra-Orthodox families, which tend to conceal VSC even more than secular families. As Prof. Gil explained, in some cases, parental religious views of gender norms interfere with medical treatment and cause severe clinical harm to children, as the following excerpt shows:

We had a girl who was … deficient in another enzyme, not 21 hydroxylase, but 11 hydroxylase … [This condition is] characterized … by hypertension. It must be diagnosed and treated promptly. She came rarely, and it was also difficult for her to come to me because she was raised as an ultra-Orthodox girl, and I am a man … and this whole situation was difficult for her. For a long time, the father came without her, only with the tests. They have two sons with XY for the same disease, and they came, but she didn’t. At one point, we didn’t monitor the blood pressure. The father claimed he was checking at home. In any case, she developed a heart condition that was partly due to hypertension and was treated for a long time with anti-hypertensive drugs and diuretics. (Prof. Gil, pediatric endocrinologist)

This severe case highlights the gap between the shared imaginings of the doctor and father regarding the girl’s condition and appropriate treatment methods versus her actual clinical state. It demonstrates the disparity between assumptions about what would benefit her and contribute to her physical and emotional well-being.

How does the data on PGT for diagnosing embryos with VSC correspond with social and medical emergencies? As mentioned, in Israel, the attitudes of medical professionals and the public toward PGT are positive (Zuckerman et al., 2020). PGT is considered preferable to TOP by religious and secular parents and viewed as a technology that allows some control over the future family’s life and health.

We collected and organized the data on PGT from two hospitals in Israel. Table 3 refers to the patients who underwent genetic counseling for future use of PGT in the context of VSC. Table 4 refers to the actual use of PGT and its implications in the context of VSC. We separated the tables to differentiate between the different stages of the diagnosis process. Of the 23 cases of couples and patients who received counseling regarding PGT to prevent VSC, 14 were ultra-Orthodox Jews, five were Muslim Arabs, and four were secular Jews. One of the reasons for the more frequent use of PGT by ultra-Orthodox Jews and Muslims is that, for religious reasons, they prefer it over facing dilemmas regarding TOP.

**Table 3:** Cases of genetic counseling regarding preimplantation genetic testing to prevent the recurrence of variations in sex characteristics

No	Mother’s age/birth year^a^	VSC diagnosis and relevant gene	Gene variants of mother (M) and father (F)	First in the family to be diagnosed Reason for PGT
1	42	CAH, CYP11B1	M = p.Arg448His F = p.Arg448His	Mother diagnosed at age eight Vulvaplasty and clitoris reduction
2	42	CAH, CYP21B	M = gene deletion F = I2 splice	One daughter with symptomatic CAH
3	35	CAH, CYP21A2	M = Val282Leu F = Val282Leu	No data available
4	34	CAH CYP21A2	M = Val282Leu, Gln319* F = Val282Leu	One son CAH, one daughter with asymptomatic CAH
5	33	CAH, CYP21A2	M = Gln319*, I2 splice F = I2 splice	One daughter with symptomatic CAH
6	32	CAH CYP21A2	M = Val282Leu, I2splice F = Gln319*	One son with asymptomatic CAH
7	30	CAH, CYP21A2	M = Gln319* F = Val282Leu, 8bpDEL	One son with symptomatic CAH
8	28	CAH, CYP21A2	M = I2 splice F = I2 splice	One son with symptomatic CAH
9	27	CAH, CYP21A2	M = Val281Leu, I2 splice F = I2 splice	One son with symptomatic CAH
10	23	CAH, CYP21A2	M = c.56G > A F = c.1066C > T	One son with symptomatic CAH. Pregnancy termination due to positive prenatal CAH testing (female)
11	21	CAH, CYP21A2	M = I2 splice F = I2 splice–deletion	Two sons with symptomatic CAH
12	1971	CAH, CYP21A2	Not available	Eldest daughter diagnosed one day after birth
13	1974	Kallmann syndrome	M = carrier of CF D1152H	Mother’s brother has Kallmann syndrome
14	1977	CAH, CYP21A2	M = NA F = Val282Leu	Father’s nephews
15	1979	CAH, CYP21A2	M = I2 splice F = Val282Leu	Eldest daughter diagnosed at age six
16	1979	CAH, CYP21A2	M = 12 splice F = ?	The daughter was diagnosed immediately after birth Prenatal US showed atypical genitalia but no further tests were conducted
17	1982	Mosaic Turner syndrome	M = 45X (50%)/ 47XXX(50%) Karyotype	After repeated miscarriages, the mother was diagnosed with Mosaic Turner syndrome
18	1984	Kallmann syndrome	No data	No data
19	1986	Androgen insensitivity syndrome	M = Sturge Weber, GNAQ c.548G > A; P.R183Q	Eldest daughter, age of diagnosis unknown
20	1987	CAH, CYP21A2	M = I2 splice F = Ile172Asn	Eldest daughter diagnosed at age four and a half In 2017, pregnancy of a fetus with CAH terminated week 14
21	1990	CAH, CYP21A2	M = Pro30Leu F = Gln319^*^	Mother with CAH. Age of diagnosis unknown
22	1994	CAH, CYP21A2	M = I2 splice F = ?	Eldest son diagnosed at age two
23	1996	CAH CYP17A1	M = homozygous F = heterozygous	Mother diagnosed with CAH. Age of diagnosis unknown

*CAH* Congenital adrenal hyperplasia, *US* Ultrasound, *VSC* Variations of sex characteristics, *PGT* Preimplantation genetic testing

^a^One hospital indicates the mother’s age at the time of genetic counseling for PGT, and another refers to the mother’s birth year. The data are organized in chronological order of birth

**Table 4:** Preimplantation genetic testing cycles and outcomes

Case Number + VSC diagnosis	Carrying-out PGT?	How many PGT cycles?	Number of embryos and diagnosis	Outcomes
1 CAH, CYP11B1	Yes	1	Three embryos tested. All affected	No record of pregnancy or follow-up treatment
2 CAH, CYP21A2	Yes	2	Data unavailable	Unavailable
3 CAH, CYP21A2	Yes	2	20 embryos tested Two carriersOne affected	Two embryos implanted. No record of pregnancy outcome
4 CAH CYP21A2	No			No record of further fertility treatment or outcomes
5 CAH, CYP21A2	Yes	1	Nine embryos tested Three carriers Five affected	No record of further fertility treatment or outcomes
6 CAH CYP21A2	Yes	3	35 embryos tested. Three wild type, seven carriers, seven affected	No record of further fertility treatment or outcomes
7 CAH, CYP21A2	No			Did not proceed with PGT. Had one natural pregnancy that resulted in an affected daughter
8 CAH, CYP21A2	No			No record of further fertility treatment or outcomes
9 CAH, CYP21A2	No			Did not proceed with PGT. Had two natural pregnancies that resulted in two affected fetuses
10 CAH, CYP21A2	Yes	1	17 embryos tested. Two carriers and one wild type	No record of further fertility treatment or outcomes
11 CAH, CYP21A2	Yes	2	11 embryos tested. Five carriers, four affected, one wild type	No record of further fertility treatment or outcomes
12 CAH, CYP21A2	No			No record of further fertility treatment or outcomes
13 Kallmann syndrome D1152H	No			Documentation of a visit to an IVF clinic and outpatient sperm aspiration procedure
14 CAH, CYP21A2	No			No record of further fertility treatment or outcomes
15 CAH, CYP21A2	Yes	No data	No data	Two babies born after PGT
16 CAH, CYP21A2	Yes	No data	No data	No record of further fertility treatment or outcomes
17 Mosaic Turner Syndrome	Yes	No data	No data	Three babies born after PGT
18 Kallmann syndrome	No			Documentation of a visit to an IVF clinic
19 AIS AR	Yes	No data	No data	Documentation of rounds of IVF
20 CAH, CYP21A2	No			No record of further fertility treatment or outcomes
21 CAH, CYP21A2	No			Documentation of the birth of twins at 24 weeks. The twins did not survive
22 CAH, CYP21A2	Yes	No data	No data	Two babies were born after PGT
23 CAH CYP17A1	Yes	No data	No data	Several attempts to become pregnant after embryo retrieval
				Record of one miscarriage
				No record of pregnancy

*AIS* Androgen insensitivity syndrome, *CAH* Congenital adrenal hyperplasia

Furthermore, CAH was the most common VSC (19 of 23 cases) for which PGT was considered (Table 3) and performed for embryo selection (Table 4). Of 19 cases of CAH, 14 included parents with children previously diagnosed with CAH (sons and daughters). Three patients had CAH, and there was one carrier couple (the father’s nephews had CAH). Another four cases in which consultation for PGT took place included one case of an older daughter with androgen insensitivity syndrome (AIS), two cases of Kallmann syndrome (in one case, the mother’s brother had Kallmann syndrome, and no data on the other case were available), and one patient with Turner syndrome (with repeated miscarriages).

In addition to atypical genitalia, bodies with VSC display a wide spectrum of sexual characteristics that diverge from female/male physical norms, including lack of gonads or partial gonads, lack of reproductive organs or combined reproductive organs, and sex chromosomes that are not 46XX or 46XY. Prenatal genetic diagnosis of VSC or PGT relies on known genetic markers, such as those associated with CAH, AIS, and Kallmann syndrome (as Table 3 shows), but many bodies with VSC have no genetic explanation. The prenatal diagnosis of differences in sex chromosomes takes place through a simple karyotype blood test between the seventh and tenth weeks of pregnancy.

Unlike the more unified perception of atypical genitalia as a medical and social emergency, a perception that motivates preventive practices, the participants’ views of differences in sex chromosomes are varied and move between the perception of bodies with these differences as highly sensitive and fragile and a rational and practical view of physical facts. This dynamic perception depends on the time of diagnosis and the interaction with parents and patients. Dr. Tal, a genetic counselor, explained the knowledge gaps and stigma surrounding different sex chromosome differences:

I remember a girl who came out XXX in amniotic fluid. The gynecologist talked to her about the possibility of terminating the pregnancy, and when she heard from me that the fetus wouldn’t have fertility problems in the future, she cried with relief because she said okay, so there is another X chromosome; it’s really not such a story. … I think that the sane approach regarding the sex chromosomes has always been here, and people decide in the end what’s right for them ... I met a couple of doctors who had a fetus with XYY, which really has nothing to do with external expression from a sexual point of view and was once thought to be more common among criminals. It’s nonsense, and you know, they said “yes” [to TOP] because they were exposed to databases, and there are studies about some kind of cognitive problem [being associated with this condition]. It’s nonsense, you know. (Dr. Tal, genetic counselor)

In contrast to Dr. Tal, Prof. Bracha believes that for some parents, sex chromosome differences may be a reason for TOP. However, these decisions depend on the time of diagnosis, as she explained:

Some terminate, and some do not. If [a fetus] is diagnosed with Klinefelter at 18 weeks and [the parents] want to terminate the pregnancy, we agree. We won’t argue with that … until week 23. … There are Ministry of Health regulations regarding the level of defects for which it is permissible to offer termination at 23 weeks. For example, there must be a 30 percent chance that there will be a severe disability and that the child will not be independent. … In Klinefelter, they will not terminate beyond week 23 if they comply with the regulations. (Prof. Bracha, gynecologist and fertility specialist)

As mentioned, estimating the frequency of such cases is difficult since there is no obligation to report data on TOP due to VSC to the Ministry of Health until the twenty-third week of gestation. Thus, despite the preference for prenatal diagnosis before week 24, in many cases, parents choose not to undergo prenatal genetic testing. How do these cases of patients of different ages reach the diagnostic stage? How do the participants perceive the genetic diagnostic process of VSC at different stages of patients’ lives?

Some participants expressed reservations regarding karyotype testing for children and teenagers. This situation mainly occurs when boys aged 16 to 17 are found to have small testicles during preparation for military service and sent for genetic testing and further examination. Sometimes geneticists refuse to perform karyotype testing for young patients, as Dr. Tal explained:

My department head treats adults, and she would receive requests for a Klinefelter test from the [military] recruiting office when they found small testicles. She would refuse or convince them why they shouldn’t have a test because what was the point? Because it will only give you some label that’s not good for you while in the army.

Many participants in this study refrained from diagnosing young adults unless they were in a relationship or facing fertility diagnosis and treatment. Dr. Dalit spoke of the following example of such a case:

This big guy came because he didn’t [need to] shave, and his hormones indicated testicular failure; the testicles weren’t functioning properly. I offered him a karyotype test to see whether it was Klinefelter, but I warned him a thousand times. “Listen,” I said, “You’re not married. Maybe get married first, and then let’s check. What do you need this information for now?” I always warn them because, at worst, he’ll ask, “What have I done? I didn’t understand. It could have been done later ...” [In the end], he did the test, and we got the result of Klinefelter. He was a … cute guy, and he went through a terrible crisis around the diagnosis, a terrible crisis. I got our social worker involved, and she talked to him. It was at the level of suicidal thoughts, not at all simple. I … identified with him because I felt his world had been destroyed. Someday, he would want to get married and have children.

In some interactions, Klinefelter syndrome is perceived as a traumatic experience, especially for patients and parents for whom biological parenting is the only option they have imagined and expected. In other interactions, Klinefelter may merely be a physical fact or difference. Amit, a genetic counselor, described a practical diagnostic process:

The last one I had was actually a guy, a boy, about to be drafted, who was sent by the recruiting office. He had Klinefelter, and … really accepted it, and so did his parents. “Okay, let’s be practical. That’s all there is. Besides that, he’s healthy. Everything is fine.” A charming guy … His father had super practical questions like, “So he shouldn’t use contraception?” … I don’t know. These are questions that you really don’t hear that much. (Amit)

What can we learn from the attempt to control the diagnostic emergency of bodies with VSC (especially those with atypical genitalia)? First, participants described a dynamic interpretive space in TOP and PGT processes. This space changes according to the dynamic interaction between patients’ physical characteristics, physicians, genetic counselors, the family, and various socio-medical-temporal factors. Hence, the diagnostic process of VSC conditions is unpredictable because it constantly evolves. Second, parental and family expectations extend beyond babies’ and children’s bodily autonomy. Third, at times, the genetic diagnosis of patients with VSC is conditional on the patient’s relationship or marital status. This conditional diagnosis assumes intimate relationships are comforting and support coping with diagnostic outcomes. The next section focuses on the dynamics of secrets and concealment in the genetic diagnostic process.

### Secrets and Concealment in the Genetic Diagnostic Process

Secrets and concealment are usually embodied through medicalization practices in the lives of people with VSC, who often do not receive relevant information and do not always provide informed consent for their diagnostic tests and treatment. Parents and doctors are reluctant to reveal the facts to children. Moreover, people with VSC experience tension and sense that something is concealed in social and familial interactions and spaces where the body is judged and examined, such as schools, clinics, and airports. How do secrets and concealment serve the preventive biomedical practices concerning VSC? How do the dynamics of secrets and concealment affect the diagnostic process?

In the various interactions between the diagnostician, the patient, the parents, and other family members, as described by our research participants, the dynamic of secrets and concealment concerning VSC creates different categories of diagnostic processes. For example, Maayan described what we term the ambivalent diagnostic process:

A woman diagnosed with a 21-hydroxylase deficiency with two mutations came for a consultation with her partner. I always try to understand the severity of the disease. … I asked them clinically and was very careful. I explained about the different types, salt wasting with virilization, without virilization, and I didn’t know what her partner knew or didn’t know… if she had chosen not to tell him … if she had had virilization and had been operated on as a child and hadn’t chosen to share it with him. It’s her personal medical information … I didn’t want to be the one to tell him or decide what he did and didn’t know, so I explained and then tried to understand what was wrong with her. It was salt wasting, and then she said she had also had virilization and that she really had undergone surgery, and then I realized that he knew. Sometimes, we are more secretive and worried about confidentiality than the patients. (Maayan)

This ambivalent diagnostic process is characterized by a hesitant, restrained, and suspicious communication style in which it is unclear who is concealing what and why.

The knowledge imparted in the ambivalent diagnostic process is measured in small steps. Maayan examined the boundary of the diagnosis, the line between disclosure and concealment of information regarding the patient’s body. For Maayan, the genital surgeries comprised the boundary of the secret, which she managed with great sensitivity and responsibility, respecting her patient’s privacy. Paradoxically, the couple had come for genetic counseling to avoid having a child with the condition that Maayan feared revealing (atypical genitalia).

Another example of an ambivalent diagnostic process was shared by Galit, a genetic counselor, who explained how she maneuvers between revealing and concealing the family secret and decides whether to approve genetic testing for the young adults involved:

Two siblings came with their mother (for genetic counseling), a 19-year-old brother, and a 21-year-old sister ... In this family, a son had been born. A circumcision was performed, and later, there was suddenly very significant salt wasting that led to the diagnosis of CAH. Several months later, surgery was performed to reconstruct the male organ into a female one. They are the (older) siblings of this daughter. … She is 12 years old, something like that. They experienced this thing 12 years ago, and from their point of view, in terms of how I perceived it from the mother, it was a very severe trauma. … I asked them what they knew about their sister, and they mentioned no details. … They knew that she needed to take pills, and that was it, nothing more. They were already [old enough to know] there had been a circumcision. They should remember that. … Suddenly, it wasn’t a boy but a girl, but it seems ... that this subject was not discussed at home. … I tried to explain to them that perhaps they should be told the truth only … when it would be relevant, when they had partners and could tell them, “I’m a carrier of [this condition], and maybe you should also be tested.” At what point do you say that? Do you want to know it before? Do you want to know that only after the wedding? … It didn’t help me. … The mother really pushed, and they did the test in the end. (Galit, genetic counselor)

Dr. Rivi also described an ambivalent diagnostic process, referring to a patient who did not want to undergo genetic testing. It was unclear whether or not he was aware of his medical history and how much his partner knew. Roni attempted to reconcile the ambivalent diagnostic process by pretending and radically altering her communication style:

It’s not just the fact that he was born with ambiguous genitalia. He also suffers from short stature. As far as I recall, there were also a few other things, and it seemed he should undergo broad genetic testing. It took a long time to convince him, but he didn’t consider it a problem. He and his wife conceived through IVF, and then it arose that he seemed slightly syndromic, so he was referred to me for genetic testing. When he came for counseling, I had already received his file from the archive, and it was so thick ... He came into the room and picked up this file. His wife also came in, and I didn’t know if he was putting on a show or not, and he didn’t know how to get out of it, and he said, “What is this, my file? Why is there so much in it? Now you see [in the file] a lot of visits over the years and not all of them were at the age of one or two that he doesn’t remember, so I think that there’s a certain element of concealment or denial ... I didn’t know how much his wife knew ... so I tried to act as if I was surprised, to go with the flow in the sense of saying “Well, let’s see what’s up then?” and not make him uncomfortable. (Dr. Rivi )

Another type of diagnostic process created by the dynamic of secrets and concealment is the conflicted diagnostic process, in which one party wishes to reveal the secret, and the other wishes to conceal it. Conflicts regarding the disclosure of the secret may delay practical decision making, lead to interruptions and delays in diagnosis and treatment, and increase stress and tension among the parties. Maayan provided an example of such an interaction:

I had a case of a 17-year-old boy referred to an endocrinologist because something was wrong with the kidneys, with diabetes, or I don’t know what, and somehow, they also sent him for a karyotype test. I don’t remember why. It turned out that he had Klinefelter, and his parents were very busy concealing it. They didn’t want to come for genetic counseling. Then they came, and they didn’t want him to come. He’s a 17-year-old boy. I wanted him to come for genetic counseling. I also said we would wait another two months until he was 18. It was my duty to call him in. They didn’t … want him to know. We told them that with Klinefelter, an attempt should be made to preserve fertility [by doing] a sperm extraction from the testicle, so how are you going to do it without him knowing? He’s 17 years old. [They replied], “We’ll sedate him.” I remember the case. His file is still here. I can’t let go of this case. They say that emotionally, this is a very problematic child because of all his other medical problems, and they don’t want to burden him with this information.

As these and other cases show, parents often assume that the secret of their child’s VSC is under their control and that by controlling it, they can influence their children’s diagnostic process and treatment. This conflicted diagnostic process can create a dangerous situation that undermines the position of medical professionals, whose responsibility is not to harm their patients. Parents who interfere to preserve secrecy may endanger their children’s health and well-being and damage the parent–child relationship. Moreover, secrets create a dimension of imagination within the relationships and communication between parents, children, and medical professionals, who may imagine that keeping the secret will prevent something negative from happening, and revealing it will create a harsh reality. Secrets create the worry that they will be revealed. Often, concerns center around the child’s future. There may be fears of suicide or losing the child’s trust in their social environments or families. Nevertheless, secrets give the illusion of control and ownership over relationships and the perception of reality.

This section also reveals how practices of secrecy and concealment are intrinsically embedded in the lives of individuals with VSC and permeate their families and future generations. The seed of bodily secrecy surrounding VSC is planted during the diagnostic process, which, while seeking to prevent such bodies from existing, paradoxically perpetuates the cycle of concealment.

### Imagining and Preventing the Existence of Fertile Bodies

Within the fertility regulation process, the dynamic between prevention and recognition of the bodily autonomy of future parents is expressed in various ways. In this section, we refer to the dynamic of the diagnostic process in the context of fertility. We focus on the interrelationships between early diagnosis of VSC and fertility and the gap between the imagined and the real in addressing the fertile body. First, we examine the data on PGT cycles in the context of the fertility regulation process (see Table 4). These data are significant because they empirically confirm the use of PGT to prevent VSC, an issue generally discussed but not studied. Next, in the context of preventive practices, we describe the use of dexamethasone to prevent the virilization of female fetuses with CAH and professionals’ perceptions of fertility among people with VSC.

As mentioned, the data available in the system cannot provide a comprehensive picture of the cases, the continuation of the fertility regulation process, or outcomes (births of babies without the gene variants of VSC). Following PGT genetic counseling, parents and patients with VSC may not continue with PGT for various reasons or may continue diagnosis and treatment elsewhere. Our data show that 13 of 23 families conducted PGT cycles. Nine of the 13 were parents with children with VSC (eight parents of children with CAH, one parent of a girl with AIS). Three of the 13 families were patients with VSC (two born with CAH and one with Turner Syndrome), and in one family (number 3), no data on a family history of CAH were available (Table 4). In addition, the data on the number of PGT cycles and how many embryos were examined and diagnosed were available for only seven families. Table 4 shows that PGT cycles were conducted from one to three times among these seven families. Between three and 35 embryos were tested, and there were no records regarding pregnancies following PGT. Of the six other families for which there is no record of the number of PGT cycles and tested embryos, the records show three cases of pregnancies following PGT, including families 15 and 22 (parents of children with CAH), who each had two children after PGT, and family 17, in which the mother is a patient with Turner syndrome who had experienced repeated pregnancy losses but gave birth to three children after PGT.

Among the ten families that did not continue with PGT, in families 7 and 9, natural pregnancies and a diagnosis of fetuses with CAH were recorded. There is no record of the birth or continued follow-up of the pregnancies. In family 21, there is a record of the premature birth of twins at 24 weeks, but they did not survive. Among two families with carriers of Kallmann syndrome, numbers 13 and 18, there is documentation of continued fertility treatments and visits to the IVF clinic. Among the five other families, there is no documentation of continued treatment. The small number of births following PGT is striking and raises questions as to whether PGT impairs birth outcomes, which it is intended to enhance.

In addition to PGT, at the beginning of pregnancy, dexamethasone, a steroid, is used to prevent the virilization of genitalia in fetuses with CAH and 46XX chromosomes. This treatment is offered during pregnancy to couples at risk of having offspring with classic CAH. Dexamethasone treatment is controversial. In a recent international study, Nowotny and colleagues state, “Current medical standards regarding Pdex [prenatal dexamethasone treatment] are lacking evidence-based guidelines on the optimal starting point, optimal duration and optimal dosing as well as standardized surveillance and follow-up at specialized centres” (Nowotny et al., 2022, p. K-22). Dreger et al. (2012) described the ethical and medical issues involved in the use of dexamethasone as a medical experiment and pointed to the lack of long-term research on its consequences for fetuses and mothers. A study conducted by Meyer-Bahlburg et al. (2012) that examined both short-term and long-term cognitive implications of dexamethasone treatment in pregnancies at risk for CAH (involving 140 children aged five to 12 and 20 participants aged 11 to 24) revealed mixed results. While short-term prenatal DEX exposure showed no significant adverse effects and even some positive effects, the findings suggest that long-term exposure in CAH girls was associated with cognitive processing delays. However, correlation analyses did not consistently support this association. These findings underscore the ongoing uncertainty surrounding DEX treatment’s cognitive impacts.

Nonetheless, many Israeli medical professionals continue prescribing dexamethasone treatment. Dr. Shay explained why he administers dexamethasone before diagnosis:

I haven’t heard about any issues. I know we are helping to provide genetic answers, and [when we do], the dexamethasone can be stopped. If there is a 25 percent chance, if both parents are carriers, but the fetus is affected, then they will terminate the pregnancy anyway, unless they say they won’t terminate the pregnancy, and then I give it to them until we receive the results of the genetic test, and if the genetic test is normal, they can stop taking it. (Dr. Shay, genetic counselor)

Often, dexamethasone is administered to prevent virilization without knowledge of whether the fetuses need this treatment in the first place. Dr. Dalit describes why:

Basically, it’s given [without knowledge of the fetus’s condition] because the genitalia are formed at an early stage, before the woman even knows she’s pregnant. … If there’s concern regarding a condition that may cause virilization in a female, you should give her steroids until you know whether it’s a male or a female. Only in one eighth of cases will you have to continue, that is, only in a situation where the fetus inherited both mutations from both parents and is a female because, in any other situation, you will not administer treatment. If [the fetus] is a male … or … does not have CAH, you don’t need treatment, so in the end, only in one eighth of the situations should the treatment be continued throughout the pregnancy. (Dr. Dalit, gynecologist and geneticist)

According to Dr. Dalit, the calculation of one eighth is as follows: Only a quarter of the fetuses of couples carrying severe CAH will have CAH. Half of those will be 46XX. Only 46XX fetuses with CAH can develop virilization, and this is what the steroids are supposed to prevent. Males with CAH should be virilized, and thus, there is no need to prevent virilization. If the drug is necessary in only one eighth of cases, the question arises whether the overall treatment is effective and for whom. What are the gaps between imagination and reality during the fertility regulation process and in fetal and maternal health following dexamethasone treatment? It is crucial to consider these questions. Nevertheless, as mentioned, our interviewees did not view the use of this drug as controversial or particularly risky. Instead, they emphasized its purpose—the prevention of virilization in female fetuses.

There are cases where adult women with various VSC conditions go for genetic diagnosis or testing during pregnancy. The interplay between early diagnosis and fertility regulation processes among people with VSC reflects gaps in knowledge, culture, and social norms regarding fertile bodies and ways of coping with difference. Prof. Hanan described his meaningful clinical interaction with a patient with Swyer syndrome (with 46XY chromosomes, a uterus, no ovaries, and female genitals):

I had a patient with Swyer syndrome. I saw her for the first time at an ultrasound scan. She was pregnant, with the help of a donor egg, but in her uterus. She … told me the story voluntarily. Otherwise, I would have had no way of knowing ... You couldn’t tell just by looking at her; you could have thought she was a big woman. … She said, “I don’t know if there are things you need to know here. I want you to know everything. You’re doing a scan for me. Check what you can. Here, these are all the data about me. Like every woman, I want the best for my fetus.”... I was very cautious. For example, before the scan, I didn’t know … what kind of vagina she had, so I gently asked her about the vaginal examination, saying, “I think I’d better do a vaginal scan. How are you with that?” (She replied), “No problem, I’ve already had countless vaginal examinations. Everything is fine.” (Prof. Hanan)

Dr. Roni also described a positive interaction with a woman who faces her physical reality without concealing her condition. This attitude is unusual in the conservative, religious sector of Israeli society, which sanctifies fertility and gender and physical norms, as Dr. Lori emphasized:

A very nice pregnant woman came. … She had a uterus and no ovaries. She became pregnant from an egg donation and was hospitalized with us … I think you always, always get a true picture of the struggles, the emotional struggles ... We live in a country where there’s no clear agreement, for example, that it’s legitimate not to have children, that it’s legitimate to have children with an egg donation or another donation. This is not the standard of care, and she was, in fact, a very beautiful example of a perfect, simple way of coping. I think life helped her in that everything went well. … She met a nice guy for whom this was not an issue, and she married. She had no difficulty sharing this. I think many people would have tended to conceal the truth because society is relatively conservative. (Dr. Lori)

Positive interactions with adults with VSC in the context of the fertility regulation process may expand knowledge and alter previous perceptions of fertile bodies. On the other hand, such interactions will diminish when early diagnoses lead to practices that prevent these bodies’ existence.

## Discussion

We have described the various practices employed to conceal or prevent the existence of bodies with VSC. These include PGT, TOP, the use of dexamethasone in cases of CAH, and secrecy. Each of these practices raises biosocial questions that undermine the perceived boundaries of the Israeli public discourse. The first is what physical and gender norms biomedical professionals and parents seek to preserve or establish. One of these norms is typical genital appearance. However, questions such as who should determine the standard clitoris size or whether vaginal construction contributes to the health of babies have not yet been answered substantively and empirically. Preventive practices aimed at bodies with VSC, especially CAH, seek to suppress and exclude any debate on genital variability.

Nevertheless, sometimes preventive practices can encourage bodily autonomy, especially for people who have faced pain and suffering due to their physical conditions and wish to prevent their children from having similar experiences. As our findings reveal, some people with CAH use PGT because it is perceived by many (doctors, patients, and parents) as a serious genetic disease that causes suffering. Further research is needed to explore the decision-making process of parents and patients with CAH who undergo PGT in Israel and other countries.

Each participant examines and re-examines their imagined or ideal body during the diagnostic process. The ability to imagine the future of people with CAH depends on people’s previous knowledge, awareness, experiences, and interactions with individuals with CAH and other VSCs. Concealment practices, the sense of emergency surrounding CAH, and preventive practices reduce the ability to recognize or imagine a positive, fulfilling life for people with VSC.

Another biosocial question is whether preventive practices prevent the recurrence of VSC or reproduce it. Does PGT technology guarantee the absence of genetic VSC? PGT may prevent specific genetic VSC conditions but cannot prevent, control, or predict all VSC’s hormonal and physiological aspects. In a way, it perpetuates the vicious circle of defining the core of (male/female) sex development.

Through the fertility regulation process, patients’ bodies are monitored, controlled, and changed through hormone therapy and other medical interventions. It is a physical and socio-medical process aimed at establishing (gendered) fertile bodies, but at the same time, it establishes physical and social experiences of uncertainty. The data show that PGT does not guarantee so-called successful pregnancies and births or babies with typical genitalia. On the other hand, the interaction between early diagnosis and fertility in the context of VSC can benefit the patient and enable future fertility, as we saw in the case of a patient diagnosed after repeated miscarriages who underwent PGT and subsequently gave birth to three children.

Secrecy and concealment are paradoxical because they cannot eliminate the presence of an actual physical body. Moreover, hiding and maintaining secrecy increases patients’ stress and sense of alienation. Those who have learned to liberate themselves from secrecy can continue with their lives and even build families. The lessons that emerge from the findings demonstrate that secrets between children, parents, and doctors in the context of VSC may endanger children’s physical and emotional health. Doctors, parents, and people with VSC should develop spaces of inclusive and beneficial communication, especially regarding patients’ needs, to gain an understanding of the basic assumptions and motivations for preventive practices.

## Footnotes

1 SRY (sex-determining region Y) is a key gene located on the Y chromosome. It activates a genetic pathway involving other genes, such as SOX9, leading to the development of Sertoli cells and other testicular tissues.
